# Exploring self-supervised learning biases for microscopy image representation

**DOI:** 10.1017/S2633903X2400014X

**Published:** 2024-11-14

**Authors:** Ihab Bendidi, Adrien Bardes, Ethan Cohen, Alexis Lamiable, Guillaume Bollot, Auguste Genovesio

**Affiliations:** 1 IBENS, Ecole Normale Supérieure PSL, Paris, 75005, France; 2 INRIA, Ecole Normale Supérieure PSL, Paris, 75005, France; 3 FAIR, Meta, Paris, 75005, France; 4 Minos Biosciences, Paris, 75005, France; 5 Synsight, Evry, 91000, France

**Keywords:** image transformations, microscopy imaging, self supervised learning

## Abstract

Self-supervised representation learning (SSRL) in computer vision relies heavily on simple image transformations such as random rotation, crops, or illumination to learn meaningful and invariant features. Despite acknowledged importance, there is a lack of comprehensive exploration of the impact of transformation choice in the literature. Our study delves into this relationship, specifically focusing on microscopy imaging with subtle cell phenotype differences. We reveal that transformation design acts as a form of either unwanted or beneficial supervision, impacting feature clustering and representation relevance. Importantly, these effects vary based on class labels in a supervised dataset. In microscopy images, transformation design significantly influences the representation, introducing imperceptible yet strong biases. We demonstrate that strategic transformation selection, based on desired feature invariance, drastically improves classification performance and representation quality, even with limited training samples.

## Impact Statement

This article investigates the impact of image transformation design in self-supervised representation learning (SSRL) for biological imaging. Our study shows how these transformations influence feature representation and model accuracy, particularly in microscopy imaging where differences are more subtle. We demonstrate that specific transformations can be strategically used to control model performance for targeted tasks. This research is of importance to computational bioimaging, offering insights on optimizing SSRL for enhanced precision and efficiency in biological research. This advancement aids in developing more accurate tools for biological exploration, fostering cost-effective scientific progress.

## 1. Introduction

In self-supervised representation learning (SSRL), a common learning objective in most approaches is for models to be trained to learn a common representation of two different transformations of the same image. The objective of SSRL is to benefit from training on a large unannotated dataset to obtain a representation that can be useful for solving downstream tasks for which one has a limited amount of annotated data. SSRL has become one of the main pillars of *Deep Learning* based computer vision approaches^(^^3^ ^,^^8^ ^–^^11^ ^,^^20^ ^,^^56^ ^)^, with performances coming close to, and sometimes going beyond supervised learning for some downstream tasks.

SSRL relies heavily on combinations of random image transformations. These transformations are used to create distorted versions of the original image with the aim of keeping the semantic content invariant. With SSRL approaches producing overall good accuracies on the downstream classification of natural images, hyper-parameter optimization of transformation parameters has added significant improvements to the overall performance of models^(^^10^ ^)^. However, further consequences of the choice of these augmentations have been only sporadically explored by the research community^(^^20^ ^,^^49^ ^)^, especially for other tasks^(^^57^ ^)^ and in other domains^(^^53^ ^)^. It is therefore unclear to what extent this choice impacts the pretraining of models at deeper levels, as well as the effects on the extracted features and the performances in other domains.

Some important questions remain unanswered. Is the accuracy for an individual class contingent upon the choice of augmentation? Can the variation of this choice increase one class’s accuracy at the expense of degrading another one? Are the features encoded into the latent representations being affected by this choice? What is the amplitude of these issues in domains other than natural images? In this paper, we report and analyze the outcomes of our experimentation to shed light on this subject. By examining the performance of various SSRL methods, while altering the selection and magnitude of transformations, we analyze and quantify their ramifications on overall performance as well as on the class-level performance of models. We subsequently seek to observe the effects of substantially varying the structure of combinations of transformations on the quality of the resulting representations. We then investigate the effects of varying selection transformations in SSRL methods when applied to microscopy images of cells, where distinctions between classes are far less discernible than with natural images, and then proceed to discuss potential avenues for improvement in the SSRL field.

Using convolution-based approaches on small to medium-scale datasets, our contributions can be succinctly summarized as follows:

- We explore the nuanced impact of transformations on performance at the class level. Our investigation reveals that the selection of transformations commonly used in Self-Supervised Learning approaches can degrade the accuracy of certain classes while improving those of others. However, we also observe that this effect is controllable and can be leveraged to beneficially manage the performance of specific classes in specific application scenarios.
- We demonstrate that, through analysis of the representations obtained from Self-Supervised trainings, the careful selection of specific combinations of transformations facilitates the optimization of models for encoding distinct features into the resulting representations. Simultaneously, this deliberate choice may result in the loss of other features, thus enabling models to be tailored for different tasks.
- We examine the implications of the choice of transformations for Self-Supervised Learning in the biological domain, where the distinction between classes is often fuzzy and subtle. Our findings illuminate the heightened importance of transformation choice within this domain, showcasing that a meticulous definition of desired features yields improvements in result quality. Moreover, our experiments demonstrate the superiority of this approach over transfer learning when dealing with small-scale datasets exhibiting domain differences.

## 2. Related work

### 2.1 Self-supervised representation learning (SSRL)

Contrastive learning approaches^(^^10^ ^,^^11^ ^,^^13^ ^,^^55^ ^)^ have shown great success in avoiding trivial solutions in which all representations collapse into a point, by pushing the original image representation further away from representations of negative examples. These approaches follow the assumption that the augmentation distribution for each image has minimal inter-class overlap and significant intra-class overlap^(^^1^ ^,^^44^ ^)^. This dependence on contrastive examples has since been bypassed by noncontrastive methods. The latter either have specially designed architectures^(^^9^ ^,^^12^ ^,^^20^ ^)^ or use regularization methods to constrain the representation in order to avoid the usage of negative examples^(^^3^ ^,^^4^ ^,^^18^ ^,^^30^ ^,^^32^ ^,^^56^ ^)^. Another line of work^(^^22^ ^,^^45^ ^)^ focuses on obtaining positive and negative examples in the feature space, bypassing the need to augment the input images with transformations.

### 2.2 Impact of image transformations on SSRL

Compared to the supervised learning field, the choice and amplitude of transformations have not received much attention in the SSRL field^(^^2^ ^,^^15^ ^,^^31^ ^,^^33^ ^)^. Studies such as^(^^52^ ^)^ and^(^^50^ ^)^ analyzed in a more formal setting the manner in which augmentations decouple spurious features from dense noise in SSRL. Some works^(^^10^ ^,^^19^ ^,^^20^ ^,^^39^ ^)^ explored the effects of removing transformations on the overall accuracy. Other works explored the effects of transformations by capturing information across each possible individual augmentation, and then merging the resulting latent spaces^(^^53^ ^)^, while some others suggested predicting intensities of individual augmentations in a semi-supervised context^(^^42^ ^)^. However, the latter approach is limited in practice as individual transformations taken alone were shown to be far less efficient than compositions^(^^10^ ^)^. An attempt was made to explore the underlying effect of the choice of transformation in the work of ^(^^37^ ^)^, one of the first works to discuss how certain transformations are better adapted to some pretext task in self-supervised learning. This study suggests that the best choice of transformations is a composition that distorts images enough so that they are different from all other images in the dataset. However favoring transformations that learn features specific to each image in the dataset should also degrade information shared by several images in a class, thus damaging model performance. Altogether, it seems that a good transformation distribution should maximize the intra-class variance while minimizing inter-class overlap^(^^1^ ^,^^44^ ^)^. Other works proposed a formalization to generalize the composition of transformations^(^^38^ ^)^, which, while not flexible, provided initial guidance to improve results in some contexts. This was followed by more recent works on the theoretical aspects of transformations,^(^^48^ ^)^ that studied how SSRL with data augmentations identifies the invariant content partition of the representation,^(^^21^ ^)^ that seeks to understand how image transformations improve the generalization aspect of SSRL methods, and^(^^57^ ^)^ that proposes new hierarchical methods aiming to mitigate a few of the biases induced by the choice of transformations.

### 2.3 Learning transformations for SSRL

A few studies showed that optimizing the transformation parameters can lead to a slight improvement in the overall performance in a low data annotation regime^(^^40^ ^,^^42^ ^)^. However, the demonstration is made for a specific downstream task that was known at SSRL training time, and optimal transformation parameters selected this way were shown not to be robust to slight changes in architecture or task^(^^43^ ^)^. Other works proposed optimizing the random sampling of augmentations by representing them as discrete groups, disregarding their amplitude^(^^49^ ^)^, or through the retrieval of strongly augmented queries from a pool of instances^(^^51^ ^)^. Further research aimed to train a generative network to learn the distribution of transformation in the dataset through image-to-image translation, in order to then avoid these transformations at self-supervised training time^(^^54^ ^)^. However, this type of optimization may easily collapse into trivial transformations.

### 2.4 Performance of SSRL on various domains and tasks

Evaluation of SSRL works relies almost exclusively on the accuracy of classification of natural images found in widely used datasets such as Cifar^(^^26^ ^)^, Imagenet^(^^16^ ^)^, or STL^(^^14^ ^)^. This choice is largely motivated by the relative ease of interpretation and understanding of the results, as natural images can often be easily classified by eye. This, however, made these approaches hold potential biases concerning the type of data and tasks for which they could be efficiently used. It probably also has an impact on the choice and complexity of the selected transformations aiming at invariance: some transformations could manually be selected in natural images but this selection can be very challenging in domains where differences between classes are invisible. The latter was intuitively mentioned in some of the previously cited studies. Furthermore, the effect of the choice of transformation may be stronger on domains and tasks where the representation is more thoroughly challenged. This is probably the case in botany and ornithology^(^^53^ ^)^ but also in the medical domain^(^^42^ ^)^ or research in biology^(^^5^ ^,^^28^ ^,^^36^ ^)^. As biology and Drug Discovery stands to benefit massively from Deep Learning and SSRL^(^^25^ ^)^, there is an ongoing focus on proving the transferability of SSRL approaches pretrained on natural images to the biological domain^(^^17^ ^)^, or leveraging the existing unlabeled biological images for biology specific SSRL pretraining with the fixed number of channels^(^^24^ ^)^ or through channel agnostic approaches^(^^6^ ^,^^23^ ^)^.

## 3. The choice of transformations is a subtle layer of weak supervision

In Section 3.1, we empirically investigate the ramifications of varying transformation intensities on the class-level accuracies of models trained using self-supervised learning techniques. Subsequently, in Section 3.2, we conduct an examination in which we demonstrate how alternative selections of transformations can lead to the optimization of the resulting representations of the model for distinct use cases. In Section 3.3, we delve into the manner in which this choice can impact representations of microscopy images, a domain where the distinction between images is highly nuanced. This is followed by an empirical analysis in Section 3.4 that illustrates how the combination of transformations chosen according to a meticulous definition of desired biological features can significantly enhance the performance of models in SSRL.

### 3.1. Transformation choices induce inter-class bias

In order to understand the ramifications of transformations on the performance of a model, we delve into the examination of the behavior of models that are trained with widely adopted SSRL techniques on the benchmark datasets Cifar10, Cifar100^(^^26^ ^)^, and Imagenet100^(^^16^ ^)^, while altering the magnitude and likelihood of the transformations. With a Resnet18, a Resnet50, and a ConvNeXt-Tiny architectures as backbones, we employ a fixed set of transformations, comprised of randomized cropping, chromatic perturbations, and randomized horizontal inversions. Subsequently, we uniformly sample a set of amplitude and probability values for each transformation, in order to create a diverse range of test conditions. Each training is repeated a number of times (three for Imagenet and five for Cifar), with distinct seed values, and the mean and standard deviation of accuracy, measured through linear evaluation over frozen weights, are computed over these five trainings for each method and each transformation value. All model training parameters, as well as the training process, are available in Supplementary Materials.

**Figure 1.**
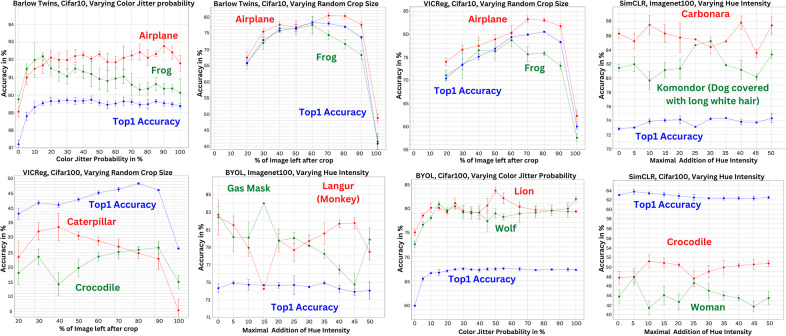
**Different transformation parameter choices induce an inter-class bias**. Inter-class Linear Probing accuracy results versus variation of a transformation parameter, for Resnet18 architectures trained with various SSRL methods on the benchmark datasets Cifar10, Cifar100, and Imagenet100. Each dot and associated error bar reflects the mean and standard deviation of three runs for Imagenet100 and five runs for Cifar with different random seeds. While overall accuracy remains relatively consistent across a range of transformation parameters, these transformations can have a subtle but significant impact on individual class performance, either favoring or penalizing specific classes. Additional comparisons are available in Supplementary Materials.

As depicted in Figure 1, we observe minimal fluctuation in the overall accuracy of each model as we slightly alter any one of the transformations. This stands in stark contrast to the class-level accuracies observed, in which we discern significant variation in the accuracy value for many classes, as we vary the parameters of transformations, hinting at a greater impact of variations in transformation parameters on the class level. Through the same figure, it becomes apparent that a number of classes exhibit distinct, and at times, entirely antithetical behaviors to each other within certain ranges of a transformation parameter. In the context of the datasets under scrutiny, this engenders a bias in the conventional training process of models, which either randomly samples transformation parameters or relies on hyperparameter optimization on overall accuracy to determine optimal parameters. This bias manifests itself in the manner in which choosing specific transformation parameters would impose a penalty on certain classes while favoring others. This is demonstrated in Figure 1 by the variation in accuracy of the Caterpillar and Crocodile classes for a model trained using VICReg^(^^3^ ^)^, as the crop size is varied (bottom left plot). The reported accuracies uncover that smaller crop sizes prove advantageous for the Caterpillar class, stimulating the model to recognize repetitive patterns and features consistent across the length of the caterpillar’s body. However, the Crocodile class doesn’t fare as well under similar conditions. This can be explained by considering the differing morphologies of the two subjects. The Caterpillar class benefits from smaller crops as the caterpillars exhibit uniformity across their body parts. Conversely, for the Crocodile class, a small crop size could potentially capture a segment like the tail, which could be misattributed to other classes, such as snakes, due to its isolated resemblance. Therefore, the choice of transformation probability or intensity directly affects class-level accuracies, an impact that may not be immediately apparent when only considering the overall accuracy.

In order to gain a deeper understanding of the inter-class bias observed in our previous analysis, we aim to further investigate the extent to which this phenomenon impacts the performance of models trained with self-supervised learning techniques. By quantitatively assessing the correlation scores between class-level accuracies obtained under different transformation parameters, we aim to measure the prevalence of this bias in self-supervised learning methods. More specifically, a negative correlation score between the accuracy of two classes in response to varying a given transformation would indicate opposing reactions for those classes to the transformation parameter variations. Despite its limitations, such as the inability to quantify the extent of bias and the potential for bias to manifest in specific ranges while remaining positively correlated in others (See Lion/Wolf pair in Figure 1), making it difficult to detect, this measure can still provide a preliminary understanding of the degree of inter-class bias. To this end, we conduct a series of experiments utilizing a ResNet18 encoder on the benchmark datasets of Cifar10 and Cifar100^(^^26^ ^)^. We employ a diverse set of state-of-the-art self-supervised approaches: Barlow Twins^(^^56^ ^)^, MoCov2^(^^11^ ^)^, BYOL^(^^20^ ^)^, SimCLR^(^^10^ ^)^, and VICReg^(^^3^ ^)^, and use the same fixed set of transformations as in our previous analysis depicted in Figure 1. We vary the intensity of the hue, the probability of color jitter, the size of the random crop, and the probability of horizontal inversion through 20 uniformly sampled values for each, and repeat each training five times with distinct seed values. We compute the Pearson, Kendall, and Spearman correlation coefficients for each pair of classes with respect to a given transformation parameter, as well as their respective p-values, and define class pairs with opposite behaviors as those with at least one negative correlation score of the three measured correlations lower than −0.3 and a *p*-value lower than 0.05. We then measure the ratio of classes with at least one opposite behavior to another class, compared to the total number of classes, in order to understand the extent of inter-class bias for a given transformation, method, and dataset.

**Figure 2.**
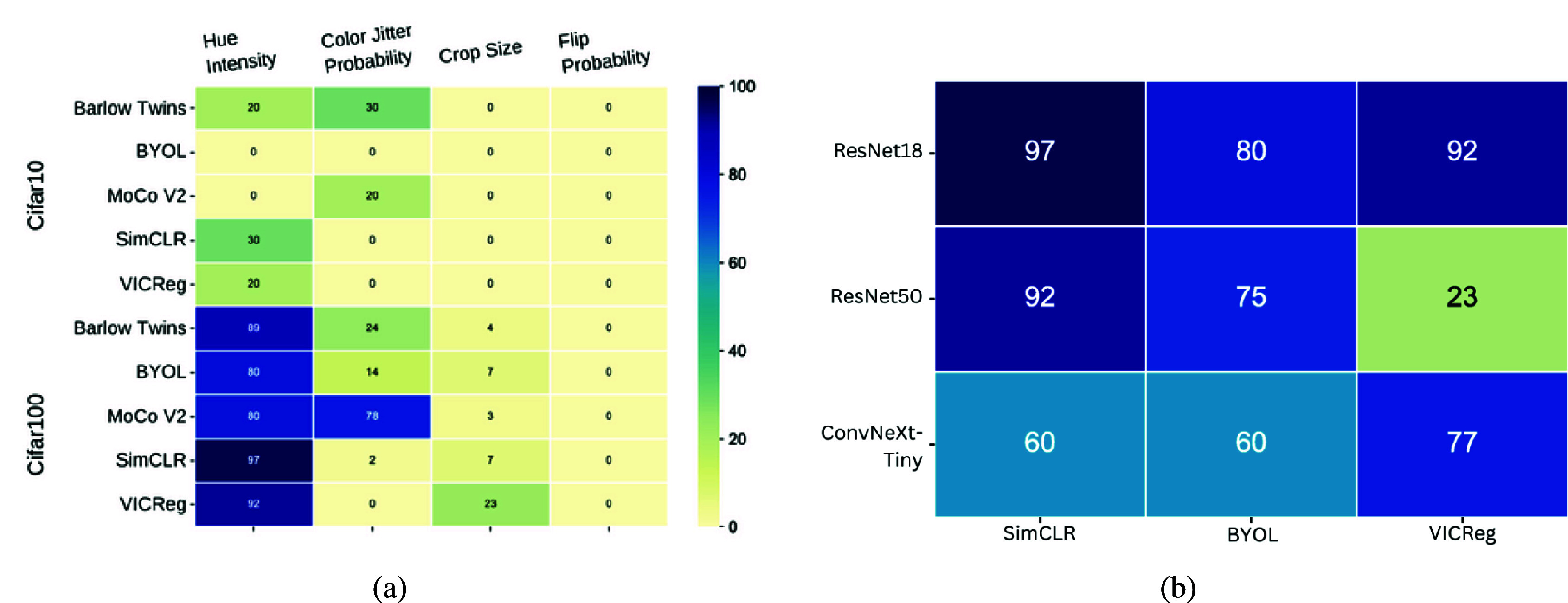
**Analyzing negative correlations in class accuracies in Cifar10 and Cifar100 datasets**. Using diverse backbones and SSRL methods, plus varying transformations, we assess the proportion of classes with negative correlations in these datasets. (a) In Cifar100 with ResNet18, more negatively correlated classes are seen, likely due to overlapping classes with increased color jitter. (b) We apply ResNet18/50 and ConvNeXt-Tiny backbones with SimCLR, BYOL, and VICReg, on Cifar100, adjusting hue intensity. The ratio of negatively correlated classes remains consistent across configurations, suggesting these patterns in (a) are independent of the SSL method and encoder architecture.

Our findings, as represented in Figure 2a, indicate that the extent of inter-class bias for the self-supervised learning methods of interest varies among different transformations. This variability is primarily due to the fact that while these transformations aim to preserve the features that define a class across the original image and its transformed versions, they can also inadvertently compromise information specific to a particular class, while favoring the information of another class. Notably, within Cifar100, a dataset encompassing a diverse range of natural image classes, we observe a significant presence of inter-class bias when manipulating hue intensity. This outcome can be attributed to the optimization of specific features through each transformation choice, which may not be optimal for certain classes. To substantiate the generality of these findings across convolution-based networks, we conduct a comparative analysis on Cifar100 using ResNet18, ResNet50, and ConvNext-Tiny as encoders, along with BYOL, SimCLR, and VICReg as the self-supervised learning approaches. By varying hue intensity, the results, as presented in Table 2b, reaffirm the consistent trend. An analysis of the number of shared classes with negative correlations between the different SSRL approaches is done in Supplementary Materials.

To investigate the potential relationship between abstract class properties and their preferred transformations, we conduct a thorough analysis of each class’s response to varying transformation parameters. We explore class accuracy behavior under distinct transformations, namely, Hue Intensity, Color Jitter Probability, and Crop Size. By computing the slope of the linear regression line that best fits the accuracy-transformation data for each class and each model, we categorize the behavior of each class accuracy as ascending, descending, or random. Simultaneously, we compute a texture analysis measure, and a Fourier transforms measure for each class in the Cifar100 dataset, as well as the spectrum of feature covariance and the intrinsic dimension using the features resulting from a ResNet model pretrained on ImageNet in a supervised manner. Using Anova and Manova correlation metrics, we then compute the correlation between these class properties and the class behaviors when varying a specific transformation.

Our results, summarized in Table 1, provide insights into the relationship between abstract image class properties and the effect of variations of transformation parameters. For instance, the Intrinsic Dimension and Texture Analysis of image classes exhibit substantial correlation with variation in Hue Intensity, implying that the intrinsic complexity and texture attributes of classes could significantly influence their response to changes in this transformation. A similar pattern is noticed with the Color Jitter Probability, albeit with a somewhat weaker correlation. Interestingly, the Spectrum of Feature Correlation shows minimal correlation with all transformations, suggesting that the covariance of class features might not significantly affect the class response to transformations. The Fourier Transform property showed mixed results, with a weak correlation with Hue Intensity but a stronger one with Crop Size, as the crop transformation can induce a varying degree of loss of signal in the image.

**Table 1.** **Correlation values between class properties and the effect of transformations on classes**. We focus on class properties such as Intrinsic Dimension, Texture Analysis, Fourier Transform, and Spectrum of Feature Covariance, and transformations such as Hue Intensity, Color Jitter Probability, and Crop Size, applied on Cifar100. Values significantly larger than 1 indicate a notable difference between behavior groups with respect to the varying transformation. Asterisks (*) denote p-values > 0.05, indicating less significant correlations

Class properties	Hue intensity	Color Jitter probability	Crop size
Intrinsic dimension	16.64	19.15	0.21*
Texture analysis	16.39	4.89	0.18*
Fourrier transform	0.71*	7	5.19
Spectrum of feature covariance	1.27	0.56*	0.97*

**Figure 3.**
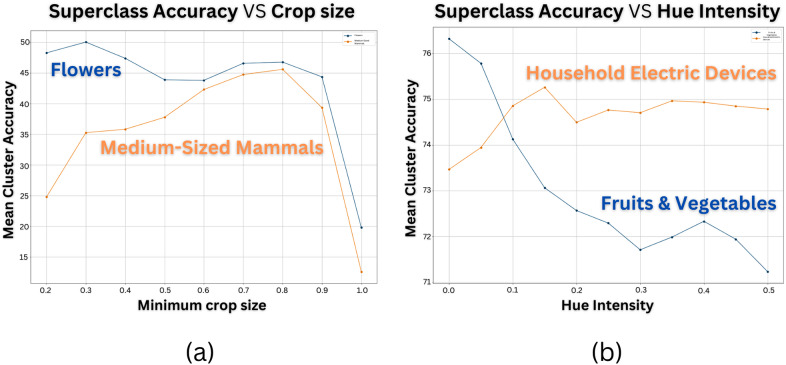
**Transformation choices influence superclass performance**. We analyze mean superclass accuracy in Cifar100 using BYOL, SimCLR, and VICReg SSRL methods, varying crop size (a) or hue intensity (b). Our observations show consistent patterns across models, highlighting distinct effects of transformation parameters on different superclasses. Each superclass has unique optimal parameters, underlining the ability of transformation selection to modulate superclass performance.

These results imply that the choice of transformations not only introduces an inter-class bias that can subtly impact performance in real-world scenarios, but also presents an opportunity to harness this bias to achieve a desired balance in class performance or optimize specific class accuracies for specific use cases. We focus in the following analysis on the coarse-grained labels of Cifar100, commonly called superclasses in the literature. As demonstrated in Figure 3, we observe that certain superclasses in the Cifar100 dataset exhibit improved recognition when specific transformation parameters are applied, when others don’t. This highlights the potential of consciously selecting and studying transformations in our training process to enhance the performance of specific class clusters or achieve a balanced performance across classes. Therefore, the careful tailoring of specific transformations and their parameters becomes crucial in preserving desired information within classes, presenting a potential avenue for improvement in training.

### 3.2. Transformation choice impacts clustering and representation information

Examining the impact of transformations on diverse tasks, with variations in transformation parameters and compositions, is crucial for comprehensively understanding their influence on the quality of the resulting representations. In our study, we specifically concentrate on the unsupervised clustering task to elucidate how the choice of transformations affects the type of encoded information. To this end, we direct our investigation towards the representations that can be attained by training encoders with different architectures (VGG11, ResNet18, ConvNeXt-Tiny) with two prominent SSRL approaches (MoCov2^(^^11^ ^)^, BYOL^(^^20^ ^)^) on the MNIST benchmark dataset^(^^29^ ^)^. Our study delves into the effects of different sets of compositions of transformations on the nature of the information embedded within the representation and its correlation to the expected intent of our tasks. For our trainings, we employ two sets of transformations. The first set, comprised of padding, color inversion, slight rotation, and random cropping, aims to maximize the intra-class overlap of digits. This is due to these transformations not affecting the digit information, and keeping it invariant in the transformed version of the images. The second set, which includes vertical flips, strong rotation, random cropping, and random erasing, enables us to investigate the representation resulting from the destruction of digit information in the transformed views, as these transformations destroy an integral part of the digit itself in the transformed version of the image. We similarly employ in additional trainings other transformation configurations sampled from each transformation set, to analyze their effects on the resulting representations. Each training iteration is repeated five times with distinct seeds, and the average of their scores is computed.

To facilitate a comprehensive comparison and evaluation of our findings, as well as to gauge the extent to which transformations can disrupt perceptual similarities between image views, we employ the Learned Perceptual Image Patch Similarity (LPIPS) metric^(^^58^ ^)^. This metric allows us to measure the perceptual similarity between views after undergoing transformations. Following the training process, we conduct a K-Means clustering^(^^35^ ^)^ with ten clusters, and a linear evaluation using the digit labels. In order to measure the efficacy of the clustering, we employ the Silhouette score^(^^41^ ^)^. This metric calculates a measure of how close each sample in one cluster is to the samples in the neighboring clusters, and thus provides a way to assess data cluster quality. Higher Silhouette scores indicate that samples are well clustered and lower scores signify that samples are incorrectly clustered. Additionally, we use the Adjusted Mutual Information score (AMI)^(^^47^ ^)^, a variation of Mutual Information that accounts for chance, providing a more robust evaluation of the clustering. The AMI score quantifies the agreement between the assigned cluster labels and the true labels, and is normalized against the expected Mutual Information to reduce its dependency on the number of clusters. A higher AMI score corresponds to a more accurate clustering with respect to the true labels. A more in-depth examination of the AMI score can be found in Supplementary Materials.

**Figure 4.**
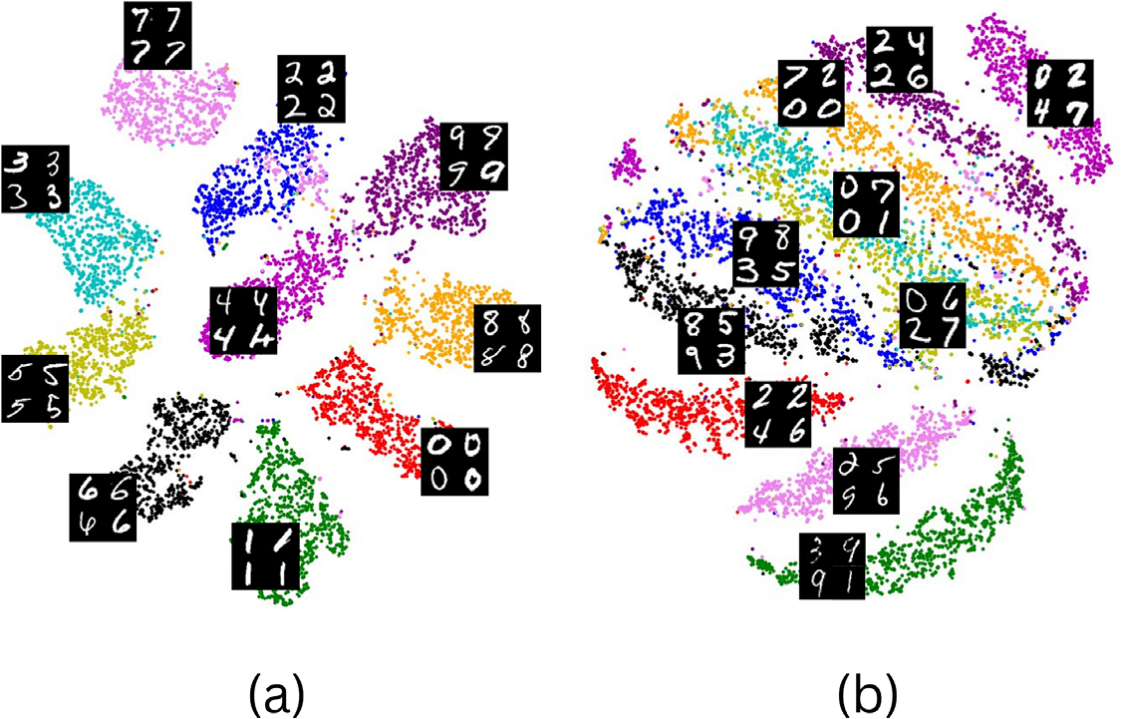
**The selection of transformations dictates the features learned during the training process, thus enabling the adaptation of a model for different tasks**. A t-SNE projection of the ten-class clustering of the MNIST dataset^(^^29^ ^)^ was performed on two representations obtained from two self-supervised trainings of the same model using MoCo V2^(^^11^ ^)^, with the sole distinction being the selection of transformations employed. One representation (a) retains information pertaining to the digit classes, achieved through padding, color inversion, rotation, and random cropping, while the other representation (b) preserves information regarding the handwriting font weight and style, achieved through vertical flips, rotation, random cropping.

As evident from the findings presented in Table 2, a progressive decline is observed in both the AMI score and the top 1 accuracy score as we transition from the initial set of basic transformations (rotation and crop) to the second set of transformations. This decline is accompanied by a notable increase in the perceptual dissimilarity between transformed image views for the second set, which is to be expected considering the highly destructive nature of the random erasing transformation in comparison to random flips. Consequently, the resulting representation manifests a substantial reduction in the information associated with digits, as evidenced by the conspicuous decline in the accuracy of digit classification, which remains unmitigated despite the implementation of supervised training during linear evaluation. Nevertheless, it is worth noting that while the AMI score experiences a more pronounced decrease, the silhouette score exhibits a slight decline. This suggests that the clusters formed by the representations resulting from the second set of transformations remain well-separated and encode meaningful information beyond mere noise. As illustrated in Figure 4, the resulting clusters from both representations demonstrate distinguishable characteristics. In particular, the clusters derived from the second set of transformations capture handwriting attributes such as line thickness and writing flow, effectively forming distinct handwriting classes where the transformations maximize intra-class variance while minimizing inter-class overlap. Additional results for alternative backbone architectures and SSRL approaches are provided in the Supplementary Materials and consistently demonstrate similar trends throughout the conducted experiments. These observations indicate that, beyond enhancing the performance of specific classes, we can selectively supervise and encode desired image features into our representations by conscientiously selecting the appropriate transformations during training. This deliberate or unconscious choice of transformations serves to improve the overall performance of the trained model for the given task.

**Table 2.** **Metrics for clustering, linear evaluation, and LPIPS** ^(^^58^ ^)^ in VGG11 models on MNIST^(^^29^ ^)^ using MoCov2^(^^11^ ^)^ and various transformations are shown. Specific transformations’ effects are examined across training configurations. The First Set, in bold, yields digit representations, while the Second Set focuses on handwriting style and thickness. Top1 Accuracy is from a Linear Evaluation, and LPIPS, using an AlexNet^(^^27^ ^)^ backbone, reflects perceptual similarity. Silhouette scores^(^^41^ ^)^ suggest good cluster quality in the second set, despite AMI scores indicating inaccurate digit cluster capture.

Transformation sets	Silhouette	AMI	Top1 Acc	LPIPS
Rotation+Crop	0.74	0.79	98.4	0.22
Rotation+Crop+Padding	0.78	0.81	99.3	0.25
**Rotation+Crop+Padding +ColorInversion (First Set)**	**0.87**	**0.83**	**99.6**	**0.33**
Rotation+Crop+Flips	0.71	0.66	96.2	0.32
**Rotation+Crop+Flips+RandomErasing (Second Set)**	**0.66**	**0.37**	**62.1**	**0.51**

### 3.3. The effect of transformations correlates with the subtlety of a domain

**Figure 5.**
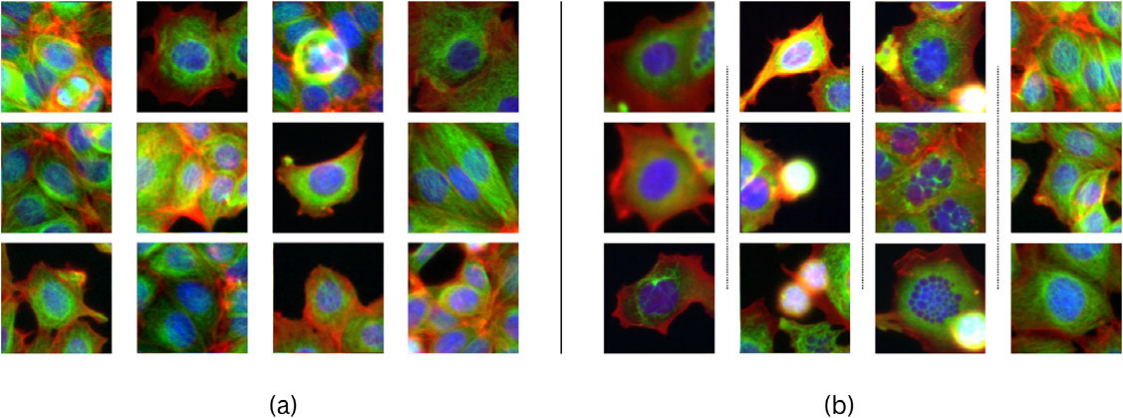
**Single cells’ genetic expression and environment** (a - untreated) cause inherent dissimilarities within conditions, challenging perturbation detection and measurement. The figure (b - high concentration Nocodazole-treated cells) shows four morphological responses to the same treatment, one resembling untreated cells (b - far right). Most lower-concentration treatments produce phenotypes visually similar to untreated cells (data not shown). Images in this dataset are centered on a cell.

With the objective of exploring the amplitude of the effect of transformations on domains characterized by inherently subtle dissimilarities among class images, we conduct experiments on microscopy images of cells under two conditions (untreated vs treated with a compound). These conditions are available from BBBC021v1^(^^7^ ^)^, a dataset from the Broad Bioimage Benchmark Collection^(^^34^ ^)^. The dataset consists of cells that demonstrate heterogeneous variability in their appearance, even when in the same condition. Notably, both the within-condition variability and the visual disparities between conditions exhibit subtle characteristics. This context poses a more demanding challenge for SSRL, as illustrated in Figure 5. In order to observe the effect of transformations in such a context, we preprocess these microscopy images by detecting all cell nuclei and extracting an 196 × 196 pixels image around each of them. We focus our study on three main compounds: Nocodazole, Cytochalasin B, and Taxol. Technical details of the dataset used and the data preprocessing performed can be found in Supplementary Material. We use a VGG13^(^^46^ ^)^ and a ResNet18 encoder architecture, with MoCov2^(^^11^ ^)^, BYOL^(^^20^ ^)^, and VICReg^(^^3^ ^)^ as the self-supervised approaches, and run two separate trainings of the model from scratch for each compound, each of the two trainings with a different composition of transformations for invariance, repeated five times with distinct seeds. We then perform a K-Means^(^^35^ ^)^ clustering (k = 2) on the inferred test set embeddings and compute the Adjusted Mutual Information score (AMI)^(^^47^ ^)^ with respect to the ground truth compound labels of the compounds data subsets (untreated vs treated with Nocodazole, untreated vs treated with Cytochalasin B, untreated vs treated with Taxol).

For each composition of transformations explored, we repeat the training five times with different seeds, and compute the average and standard deviation of the AMI score (technical details of the model training can be found in Supplementary Material). Table 3 displays the AMI scores achieved by using two different compositions of transformations in training, compared to the AMI score of clustering on representations achieved with a pretrained model trained on ImageNet with supervision. By replacing slight random cropping and resizing, a transformation used in most existing self-supervised approaches, with very strong random rotations (360°) of the image, we report a significantly higher mean AMI score, which shows that the model using random rotations, as is commonly used in the biological domain, is able to learn representations that better separate untreated from compound treated cells, including cells displaying subtle differences unnoticeable by the naked eye. Inversely, random cropping was destructive to the sought-out information in this case, as the cropped images can miss out on relevant features in the sides of the cell. In contrast to the effects of transformations reported on the overall accuracy of datasets with less subtle class differences, as discussed in Section 3.1, the markedly greater impact observed on this specific type of data implies that transformations can exert a more substantial influence on the learning of more effective representations, which capture the full range of image variability within datasets characterized by subtle distinctions between classes. By optimizing the selection of transformations for specialized goals on such datasets, our preliminary analysis shows that competitive performance comparable to models pretrained with supervision can be achieved, even in the context of a relatively small-scale dataset.

**Table 3.** **The results of the adjusted mutual information score** ^(^^47^ ^)^ **obtained for two sets of transformations, with different SSRL approaches and backbones**, through the mean of five training runs for each, compared to each other and to the AMI score achieved on the representations of pre-trained models (Resnet 101 and VGG16) trained with supervision on ImageNet, and applied on the dataset subsets containing Nocodazole, Cytochalasin B and Taxol. The selection of the pretrained models width is studied in Supplementary Materials. Both sets of transformations comprise random rotations, affine transformations, color jitter, and flips, with the first set including an additional random cropping, and resulting in a mediocre AMI score, and the second set applying random rotations and resulting in a significantly higher score.

Transformations	SSRL approach	Backbone	Nocodazole	Cytochalasin B	Taxol
First set :	MoCo v2	VGG13	0.19	0.27	0.16
		ResNet18	0.17	0.25	0.15
	Byol	VGG13	0.21	0.28	0.19
		ResNet18	0.2	0.25	0.17
	VICReg	VGG13	0.19	0.26	0.2
		ResNet18	0.16	0.25	0.21
Second set :	MoCo v2	VGG13	0.37	0.45	0.38
		ResNet18	0.33	0.42	0.31
	Byol	VGG13	0.38	0.48	0.41
		ResNet18	0.35	0.44	0.34
	VICReg	VGG13	0.38	0.44	0.36
		ResNet18	0.34	0.43	0.3
Pretrained models on ImageNet		VGG16	0.34	0.55	0.36
		ResNet101	**0.39**	**0.57**	**0.43**

**Figure 6.**
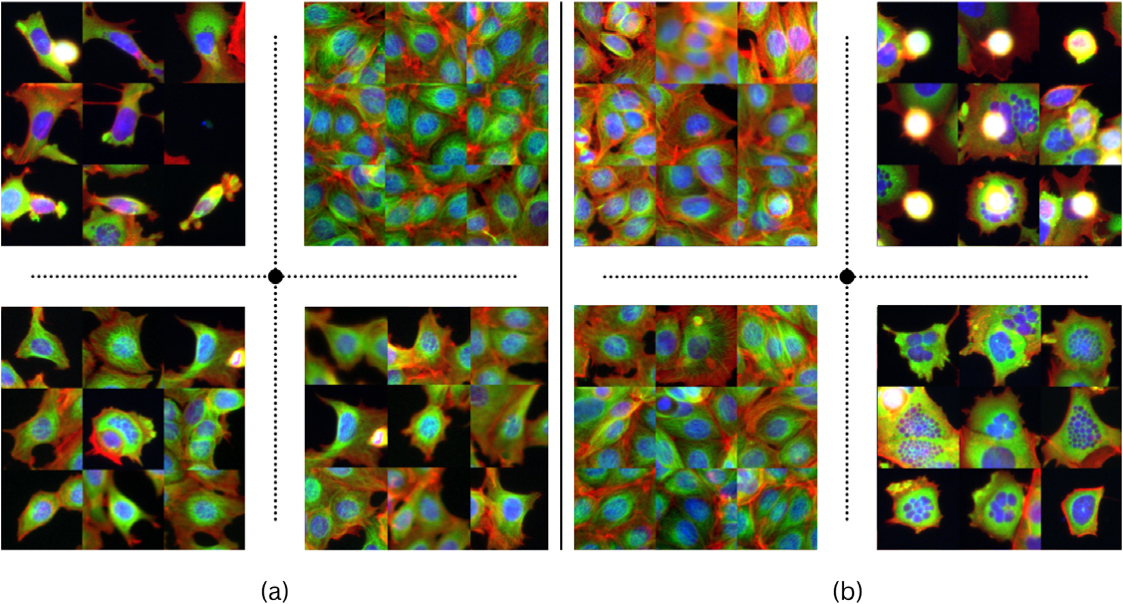
**K-Means (k = 4) clustering on Nocodazole data subset** (see Supplementary Material), using VGG13 and MoCov2 with different augmentations, aims to categorize cells’ morphological responses. Images nearest to each cluster’s centroid are based on Euclidean distance in representations. Clusters in (a), formed with color jitter, flips, rotation, affine transformation, and random cropping, focus on cell quantity per image.Clusters in (b), from rotations, center cropping, color jitter, and flips, consider specific phenotypes. Transformation details are in Supplementary Material.

Beyond clustering into two conditions, we wonder what combination of transformations could lead to a proper clustering of cell phenotypes (or morphology). We explore different compositions of transformations in additional experiments with the same VGG13^(^^46^ ^)^ architecture and MoCov2^(^^11^ ^)^ loss function. We then apply K-Means^(^^35^ ^)^ clustering (k=4) on the representations obtained from the test set. As observed in Section 3.2, different compositions of transformations can lead to very different clustering results. This is further confirmed in the microscopy domain and well illustrated by observing the Nocodazole treatment (Figure 6) where the composition of color jitter, flips, rotation, and affine, in addition to random crops, results in clustering images by the number and size of cells, rather than by morphological features (*Figure 6 left*). We perform a training where affine transform and random crop are replaced by a center crop that preserves 50% of the image around the central cell. The latter resulted in four clusters where two out of the three cell phenotypes were detected. However, it also had the effect of splitting untreated cells into two different clusters (*Figure 6 right*). This aligns with the findings presented in Section 3.2, as the diverse clustering outcomes mirror the distinct transformation approaches taken to encode the intrinsic information embedded within the images of this dataset. The presence of subtle variations within this dataset underscores the heightened sensitivity to the selection of transformations, which amplifies the multitude of potential representations accordingly. Altogether, engineering a combination of transformations in this context represents a somewhat weak supervision that can become a silent but strong bias or, alternatively, can be leveraged as a powerful tool to achieve a desirable result on a specialized task.

### 3.4. A delineation of desired features enhances task-specific representations.

Upon examining the cell distributions in Figure 5, which contrasts untreated and Nocodazole-treated cells, we observe distinct cellular responses. These observations suggest that accurately distinguishing cellular responses requires analysis of both intra- and intercellular changes. Accordingly, our analysis targets the morphological characteristics of cellular components and, to a lesser extent, the spatial relationships between neighboring cells, this being our definition of the sought-out phenotypes. To test our hypothesis, we reconduct training experiments using three different compounds: Nocodazole, Taxol, and Cytochalasin B.

To preserve and emphasize these identified features during training, we carefully select a set of image transformations. These include Affine transformations, color jitter, random rotations, and center cropping aimed at focusing on the central cell, facilitated by our preprocessing steps. This selection of transformations allows the model to focus on and learn critical morphology-related features of the cells. To further highlight intercellular relationships, we utilize random cropping, which adjusts the focus beyond the central cell.

However, it is crucial to note that certain combinations of transformations, such as random cropping and center cropping, can conflict, potentially leading to the loss of important features. This conflict underscores the complexity of selecting appropriate transformations that both capture and preserve essential cellular information.

Consequently, we define two distinct transformation compositions, each corresponding to the compositions depicted in Figure 6, and keep both separated in two independent SSRL losses of the same SSRL approach. Subsequently, we train a model to minimize the weighted sum of these two losses on each of the compound data subsets. Following training, we perform K-Means clustering (k = 4) on the resulting representations for the Nocodazole and Taxol subsets, and K-Means clustering (k = 2) on the resulting representation for the Cytochalasin B subset. Further details on the parameters of the weighted sum are available in Supplementary Materials.

**Figure 7.**
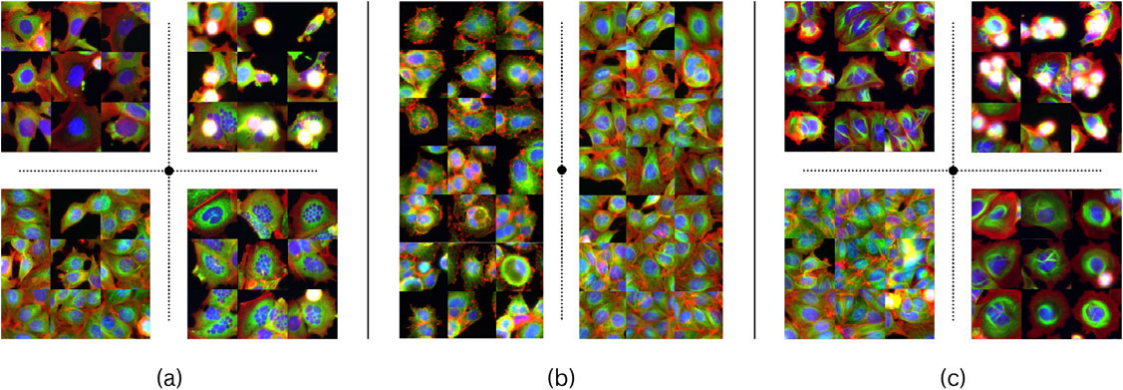
**The clustering results were achieved through the utilization of two MoCo v2 losses** ^(^^11^ ^)^ **with a VGG13 backbone, each with a distinct set of transformations**, on the Nocodazole (a), Cytochalasin B (b), and Taxol (c) image treatment subsets. One loss employs color jitter, flips, rotation, affine transformation, and random cropping, while the other uses rotations, center cropping, color jitter, and flips. The clustering results demonstrate that the phenotypes of each subset are clearly separated and represented in each cluster, as evidenced by the images closest to its centroïd.

In the subsequent results in Figure 7 we can observe that we successfully separate all cell phenotypes/morphological alterations obtained after each of the three compound treatments, from each other and from untreated cells, which validates qualitatively our hypothesis of the features of interest. By performing another K-Means (k = 2) on the same representations, we also report AMI scores to analyze the separation level of the compound-treated cells from untreated cells in the representation space. The resulting scores in Table 4 can be considered quite high in this context where treated cells can look like untreated cells, vastly surpassing previous trainings with separate compositions of transformations in Table 3, as well as surpassing the performance of pretrained models even with a small scale dataset.

**Table 4.** **AMI score comparison in K-Means (k = 2) clusterings** of models trained with two SSRL losses versus an ImageNet pre-trained encoder on Nocodazole, Cytochalasin B, and Taxol treated cell subsets. One SSRL loss uses color jitter, flips, rotation, affine transformation, and random cropping; the other, rotations, center cropping, color jitter, and flips. Careful selection of transformation sets, tailored to desired features, enhances clustering performance in self-supervised training over supervised pre-trained models, even in small datasets.

Transformations	SSRL approach	Backbone	Nocodazole	Cytochalasin B	Taxol
Weighted combination of sets	MoCo v2	VGG13	0.51	0.66	0.52
		ResNet18	0.46	0.63	0.47
	Byol	VGG13	0.51	0.64	**0.54**
		ResNet18	0.47	0.61	0.48
	VICReg	VGG13	**0.55**	**0.67**	0.51
		ResNet18	0.5	0.63	0.45
Pretrained models on ImageNet		VGG16	0.34	0.55	0.36
		ResNet101	0.39	0.57	0.43

**Figure 8.**
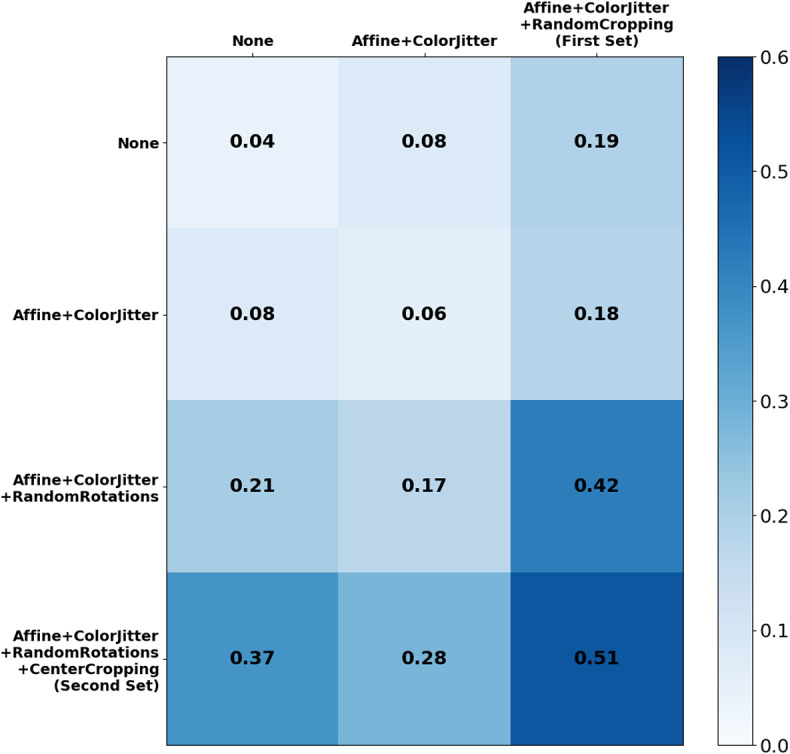
**Ablation study on Adjusted Mutual Information (AMI) scores** with progressive transformation integration, using MoCo v2 SSRL and VGG13. The first set of transformations, especially random rotations, notably improves the score and representation. Including center cropping, focusing on cellular center, further enhances results.

For a more comprehensive analysis of the results in Table 4, we conduct a more systematic examination of the interplay and impact of each of the two compositions of transformations on the combined performance of the weighted sum of losses, by modifying each combination before incorporating it with the other transformation during the Nocodazole training. The results presented in Figure 8 reveal substantial variations in the scores and, consequently, the resulting feature representations among different parts of the transformation combinations. Particularly noteworthy is the enhancement in performance when gradually incorporating transformations into the second set, which focuses on rotation-invariant features and the cellular center, while keeping the first set fixed. In contrast, the reverse process of gradually adding transformations to the first set, while keeping the second set constant, does not yield iterative improvements. This observation suggests that our defined features for this task accurately capture the significance of the cellular center, which remains invariant under rotation transformations or center cropping, in effectively separating treated cells from untreated ones. Furthermore, it demonstrates that the surrounding cells also play a role, albeit to a lesser extent, in achieving optimal representation for the task. This signifies that moving beyond rotations and center cropping, to different transformations that leverage the subtle information residing in inter-cellular interactions further improves representations for biologically relevant tasks. These findings underscore the potential of more intricate transformation manipulations, beyond single parameter modifications, to yield superior representations that significantly enhance task performance. However, identifying the optimal combination necessitates a rigorous definition of the sought-after features, which, although label-free, provides a form of weak supervision.

## 4. Conclusion

In this work, we delve into the impact of transformation choices on convolution-based approaches, specifically focusing on small to medium-scale datasets. Our experiments demonstrate that the selection, magnitude, and combination of transformations significantly influence the efficacy of self-supervised representations. Notably, the choice of transformations not only introduces inter-class bias but also offers a powerful tool for controlling and balancing class performances. Our main conclusion advocates for defining transformation sets based on the specific features sought in each task, such as the relationship of the cell to surrounding cells, rather than relying solely on predefined choices like rotations and center crops. Moreover, by carefully selecting and defining transformations to optimize the encoding of specific features, we achieve improvements in task-specific performance. Importantly, our findings emphasize the amplified consequences of transformation choices in Microscopy Imaging, a domain characterized by fuzzy or less visually distinguishable class differences, surpassing the performance of pretrained models on small-scale datasets. Altogether, some of the results can be understood somewhat intuitively. If one erases color from a car dataset, a deep network might not find enough correlated information to be able to classify cars on the basis of their original color. Thus the question: what is a good representation? In scenarios where massive datasets are not readily available, the correct answer is that it depends on the desired task. Although the initial goal of SSRL was to circumvent such circumstances, our findings in this particular context reveal the efficacy that can be achieved by judiciously selecting an appropriate combination of transformations, informed by a profound comprehension of the most salient features.

This study acknowledges several limitations, such as the utilization of small to medium-scale datasets and convolution-based approaches. Future research should consider examining larger datasets and exploring the potential benefits of combining them with transformers. Furthermore, expanding the analysis to include downstream task performance could provide a more comprehensive understanding of the impact of transformations. A promising perspective lies in the potential use of informed transformation choices to fine-tune foundational models for specific tasks, even in the absence of labeled data. Overall, this study contributes valuable insights and suggests promising avenues for future investigations in the field of transformation learning within deep learning frameworks.

Our research underscores the importance of thoughtful transformation selection in self-supervised learning, encouraging a more discerning approach to hyperparameter choice. This may inspire a shift from automatic or blind selection towards a more principled understanding of augmentations, potentially leading to more robust and nuanced model performances across diverse domains. We see no significant negative ethical implications at this time.

## Supporting information

Bendidi et al. supplementary material Bendidi et al. supplementary material

## Data Availability

All datasets used are freely available to use: ImageNet (https://www.image-net.org), Cifar (https://www.cs.toronto.edu/kriz/cifar.html), MNIST (http://yann.lecun.com/exdb/mnist/), BBBC021 (https://bbbc.broadinstitute.org/BBBC021).

## Supplementary material

The supplementary material for this article can be found at https://doi.org/10.1017/S2633903X2400014X.
