# Supplementary material for: Exploring self-supervised learning biases for microscopy image representation
Source: Biol Imaging. 2024 Nov 14;4:e12. doi: 10.1017/S2633903X2400014X (PMC11704125; doi:10.1017/S2633903X2400014X)
Supplement: Bendidi et al. supplementary material [file S2633903X2400014Xsup001.pdf]

Biological Imaging (2023), 1: 1–9
doi:10.1017/blg.2023.xx

RESEARCH ARTICLE

Appendix : Exploring Self-Supervised Learning Biases for Microscopy Image Representation

Ihab Bendidi[1,4], Adrien Bardes[2,3], Ethan Cohen[1,5], Alexis Lamiable[1], Guillaume Bollot[5] and Auguste Genovesio[1*]

[1]IBENS, Ecole Normale Supérieure PSL, Paris, 75005, France
[2]INRIA, Ecole Normale Supérieure PSL, Paris, 75005, France
[3]FAIR, Meta, Paris, 75005, France
[4]Minos Biosciences, Paris, 75005, France
[5]Synsight, Evry, 91000, France
*Corresponding author. Email: auguste.genovesio@ens.psl.eu

Received: ; **Revised:**; **Accepted:**

Keywords: Self Supervised Learning, Image Transformations, Microscopy Imaging

A. Appendix. Datasets

We perform the experiments mentioned in Section 3.3 on microscopy images available from BBBC021v1 (2), a dataset from the Broad Bioimage Benchmark Collection (16). This dataset is composed of breast cancer cells treated for 24 hours with 113 small molecules at eight concentrations, with the top concentration being different for many of the compounds through a selection from the literature. Throughout the quality control process, images containing artifacts or with out of focus cells were deleted, and the final dataset totalled into 13,200 fields of view, imaged in three channels, each field composed of thousands of cells. In the cells making up the dataset, twelve different primary morphological reactions from the compound-concentrations were identified, with only six identified visually, while the remainder were defined based on the literature, as the differences between some morphological reactions were very subtle. We perform a simple cell detection in each field of view, in order to crop cells centered in (196x196px) images. We then filter the images by compound-concentration, and keep images treated by Nocodazole at its 4 highest concentrations. These compound-concentrations result in 4 morphological cell reactions, for which we don't have individual labels for each cell image. We sample the same number of images from views of untreated cells, totalling into a final data subset of 3500 images, which we split into 70% training data, 10% validation data and 20% test data. We repeat the same process for the compounds Taxol and Cytochalasin B, each containing 4 and 2 morphological reactions respectively, to result in data subsets of sizes 1900 and 2300 images, respectively.

B. Appendix. Model training

B.1. Study of inter-class bias in self supervised classification

For results in Section 3.1 and Table 2, we run several trainings of 12 SSRL methods (1,3,4,6–8,10,11,15,19,20) on Cifar10 and Cifar100 (12) with a Resnet18 architecture, with a pretraining of 1000 epochs without labels. We use the same transformations with similar parameters on all approaches, namely a 0.4 maximal brightness intensity, 0.4 maximum contrast intensity, 0.2 maximum saturation intensity, all with a

© The Authors(s) 2023. Published by Cambridge University Press. This is an Open Access article, distributed under the terms of the Creative Commons Attribution licence (http://creativecommons.org/licenses/by/4.0/), which permits unrestricted re-use, distribution, and reproduction in any medium, provided the original work is properly cited.

fixed probability of 80%. With a maximal hue intensity of 0.5, we vary the hue probability of application between 0% and 100% by uniformly sampling 20 probability values in this range. We use stochastic gradient descent as optimization strategy for all approaches, and through a line search hyperparameter optimization, we use a batch size of 512 for all approaches except Dino[5] and Vicreg[1] for which we use a batch size of 256. Following the hyperparameters used in the literature of each approach, we use a projector with a 256 output dimension for most methods, except for Barlow Twins[19], Simsiam[8], Vicreg[1] and Vibcreg[15], for which we use a projector with a 2048 output dimension, and DeepclusterV2[3] and Swav[4] for which we use a projector with a 128 output dimension. For some momentum based methods (Byol[11], MocoV2+[7], NNbyol[10] and Ressl[20]), we use a base Tau momentum of 0.99 and use a base Tau momentum of 0.9995 for Dino[5]. For Mocov2+[7], NNCLR[10] and SimCLR[6], we use a temperature of 0.2.

We train the Barlow Twins[19] based model with a learning rate of 0.3 and a weight decay of 10^{-4}, and Byol[11] as well as NNByol[10] with a learning rate of 1.0 and a weight decay of 10^{-5}. We train DeepclusterV2[3] with a learning rate of 0.6, 11 warmup epochs, a weight decay of 10^{-6}, and 3000 prototypes. We train Dino with a learning rate of 0.3, a weight decay of 10^{-4}, and 4096 prototypes, while we train MocoV2+[7] with a learning rate of 0.3, a weight decay of 10^{-4} and a queue size of 32768. For NNclr[10], we use a learning rate of 0.4, a weight decay of 10^{-5}, and a queue size of 65536. We train ReSSL[20] with a learning rate of 0.05, and a weight decay of 10^{-4}, while we train SimCLR[6] with a learning rate of 0.4, and a weight decay of 10^{-5}. For Simsiam[4], we use a learning rate of 0.5, and a weight decay of 10^{-5}, and use for Swav[4] a learning rate of 0.6, a weight decay of 10^{-6}, a queue size of 3840, and 3000 prototypes. We use for Vicreg[1] and Vibcreg[15] a learning rate of 0.3, a weight decay of 10^{-4}, an invariance loss coefficient of 25, and a variance loss coefficient of 25. We use a covariance loss coefficient of 1.0 for Vicreg[1] and a covariance loss coefficient of 200 for Vibcreg[15]. We perform linear evaluation after each pretraining for all methods, through freezing the weights of the encoder and training a classifier for 100 epochs. We use 5 different global seeds (5, 6, 7, 8, 9) for each hue intensity value, and compute the mean top1 accuracy resulting from the linear evaluation, using each of the 5 different experiences. Each training run was made on a single V100 GPU. On ImageNet100[9], we train a Resnet18 encoder with BYOL[11], MoCo V2[7], VICReg[1] and SimCLR[6], using a batch size of 128 for 400 epochs. We use a learning rate of 0.3 and a weight decay of 10^{-4} for MoCo V2 and VICReg, and a weight decay of 10^{-5} and learning rates of 0.4 and 0.45 for SimCLR and BYOL respectively. We repeat each experience three times with three global seeds (5,6 and 7) and compute its mean and standard deviation. We repeat the same experiment with the same parameters on ResNet50 and ConvNeXt-Tiny.

For the results in Figures 1 and 2, we reuse the same training hyperparameters for Barlow Twins[19], Moco V2[7], BYOL[11], SimCLR[6] and Vicreg[1], and uniformly sample 10 values in the range of [0;0.5] for the maximal hue intensity, with a fixed 80% probability. We run different experiments for the 5 global seeds for each hue intensity, and compute their mean and standard deviation. We perform the same process while fixing maximal hue intensity to 0.1, and varying its probability by uniformly sampling 20 probability values in the range [0;100]. We repeat a similar process for the random cropping and horizontal flips, by sampling 8 values uniformly in the range of [20;100] of the size ratio to keep of the image, and sampling 20 values uniformly in the range of [0;100] for the probability of application of horizontal flips.

B.2. MNIST Clustering

For the displayed clustering results in Figure 4, we use a VGG11[17] architecture, with a projector of 128 output dimension, trained using a MocoV2+[7] loss function on Mnist[14] for 250 epochs. We use an Adam optimizer, a queue size of 1024, and a batch size of 32. We set temperature at 0.07, learning rate at 0.001, and weight decay at 0.0001. We run two trainings with two separate sets of compositions of transformations, each run on a single V100 GPU, and perform a Kmeans (K=10) clustering on the resulting representations of the test set. For the digit clustering, we use a composition of transformations

composed of a padding of 10% to 40% of the image size, color inversion, rotation with a maximal angle of 25°, and random crop with a scale in the range of [0.5;0.9] of the image, and then a resizing of the image to 32x32 pixels. For the handwriting flow clustering, we use a composition of transformations composed of horizontal & vertical flips with an application probability of 50% each, rotations with a maximal angle of 180°, random crop with a scale in the range of [0.9;1.1], and random erasing of patches of the image, with a scale in the range of [0.02;0.3] of the image and a probability of 50%. We perform linear evaluation by training classifiers on the frozen representations of the trained models, in order to predict the digit class, and evaluate using the top1 accuracy score. For results on Table 4 and Figure 4, we use ResNet18 and ConvNeXt-Tiny architectures with BYOL and MoCov2 ass SSRL approaches. We use for BYOL a learning rate of 0.01 and a weight decay of 10^{-5}, with a projector with a 256 output dimension. We then use the same parameters and augmentations as previous trainings.

B.3. Clustering evaluation with the AMI Score

We use the adjusted mutual information (AMI)(18) in Sections 3.3 and 3.4 to evaluate clustering quality, and to measure the similarity between two clusterings. It is a value that ranges from 0 to 1, where a higher value indicates a higher degree of similarity between the two clusterings. This score holds an advantage over clustering accuracy(13) in one main aspect, being that the clustering accuracy only measures how well the clusters match the labels of the true clusters, and does not take into account the structure within the clusters, such as heterogeneity of some of the clusters. This is unlike the AMI score, which takes into account both the structure between the clusters and the structure within the clusters, by measuring the "agreement" between the groupings of a predicted cluster and the groupings of the true cluster. If both clusterings agree on most of the groupings, then the AMI score will be high, and inversely low if they do not.

The AMI score can be computed with the formula :

$$AMI(X,Y) = \frac{MI(X,Y) - E(MI(X,Y))}{max(H(X),H(Y)) - E(MI(X,Y))}$$

Where $MI(X,Y)$ is the mutual information between the two clusterings, $E(MI(X,Y))$ is the expected mutual information between the two clusterings, $H(X)$ is the entropy of the clustering X, and $H(Y)$ is the entropy of the clustering Y. Mutual information (MI) is a measure of the amount of information that one variable contains about another variable. In the context of AMI, the two variables are the clusterings X and Y. $MI(X,Y)$ is a measure of to what extent the two clusterings are related to each other. Entropy is a measure of the amount of uncertainty in a random variable. In the context of AMI, the entropy of a clustering ($H(X)$ or $H(Y)$) is a measure of how much uncertainty exists within the clustering. Expected mutual information ($E(MI(X,Y))$) is the average mutual information between the two clusterings, assuming that the two clusterings are independent.

The adjusted mutual information (AMI) is calculated by first subtracting the expected mutual information ($E(MI(X,Y))$) from the actual mutual information ($MI(X,Y)$). This results in a measure of of the extent of the relationship between the two clusterings beyond what would be expected by chance. This value is then divided by the difference between the maximum possible entropy ($max(H(X),H(Y))$) and the expected mutual information ($E(MI(X,Y))$). Normalization of the result is achieved through this process, ensuring that it is always between 0 and 1. Figure 1 shows the results of a clustering achieved on Nocodazole vs untreated cells, with the AMI score computed after randomisation of the ground truth labels, in contrast to clustering results achieved without randomizing the labels.

B.4. Cellular Clustering

For the results in Sections 3.3 and 3.4, we use a VGG13(17) architecture, trained using a MocoV2+(7) loss function on the data subsets of the microscopy images available from BBBC021v1(2), presented in Section A, with a batch size of 128, for 400 epoch. We use an Adam optimizer, a queue size of 1024,

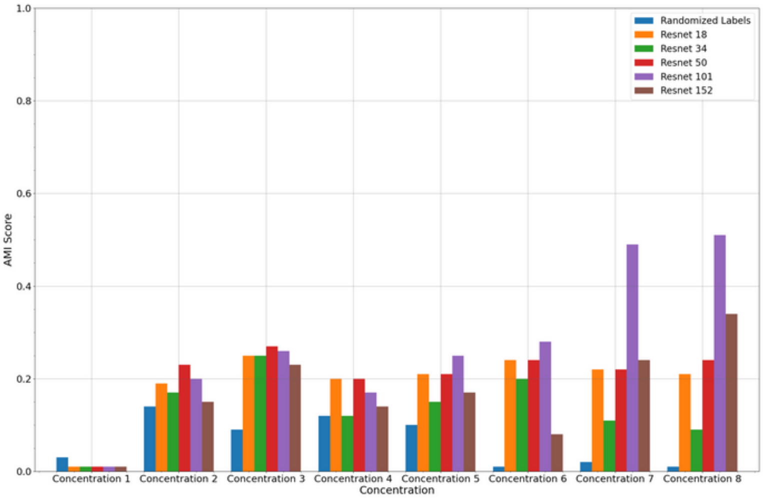

Figure 1. *We perform a Kmeans clustering (K=2) on the Nocodazole and untreated images using Resnet models of various sizes, and evaluate them using the AMI score*[(18)] *on a similar number of randomly sampled images of each concentration of Nocodazole and a similar number of untreated cell images. We observe that Resnet101 outperforms the other Resnet model sizes. We also perform an experiment to better interpret the AMI scores achieved, by randomizing the labels and performing a clustering with Resnet101, and reporting the AMI score of the clustering compared to the randomized labels.*

and set temperature at 0.07, learning rate at 0.001, and weight decay at 0.0001. Each training is made on a single V100 GPU. We perform a Kmeans (K=2) on the resulting representations of the test set of the data subsets of Nocodazole, Cytochalasin B and Taxol, and evaluate the quality of the achieved clusters compared to the ground truth using the AMI score[(18)].

Table 1. *Comparison of classes with significant negative correlations under variations of Hue Intensity for Linear Evaluation and Fine-tuning phases. The table displays the number of classes with statistically significant negative correlations (p-value < 0.05) for both Linear Evaluation and Fine-tuning under different SSL methodologies, SimCLR, BYOL, and VicReg, all with ResNet18 as the backbone. The last row represents the percentage of shared classes between Linear Evaluation and Fine-tuning that exhibited the same behavior trend (ascending, descending, or random).*

Methodology	Simclr	BYOL	VicReg
Resnet18 + Linear Evaluation	97	80	92
Resnet18 + Finetuning	96	100	92
Class Behavior match	45%	52%	53%

In Table 3, we achieve the first AMI result through usage of an affine transformation composed of a rotation with an angle of 20°, a translation of 0.1 and a shear with a 10° angle, coupled with color jitter with a brightness, contrast and saturation intensity of 0.4, and a hue intensity of 0.125, with a 100% probability, as well as random cropping of the image with a scale in the range of [0.9;1.1] and resizing to original image size of 196x196 pixel. For the second row result, we use an affine transformation composed of a rotation with an angle of 20°, a translation of 0.1 and a shear with a 10° angle, coupled with color jitter with a brightness, contrast and saturation intensity of 0.4, and a hue intensity of 0.125, with a 100% probability, and a random rotation with a maximal angle of 360°. The results achieved

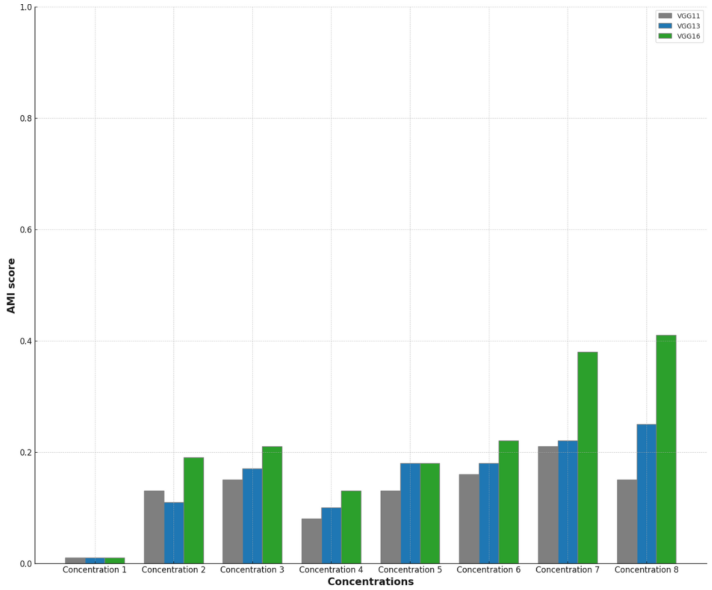

Figure 2. *We perform a Kmeans clustering (K=2) on the Nocodazole and untreated images using VGG models of various sizes, and evaluate them using the AMI score*[18] *on a similar number of randomly sampled images of each concentration of Nocodazole and a similar number of untreated cell images. We observe that VGG16 outperforms the other VGG model sizes.*

through a Resnet101, are achieved by performing a Kmeans (K=2) on the representations achieved on the compound subsets using the Resnet101, and evaluating the cluster assignment quality compared to ground truth using the AMI score[18]. The choice of Resnet101 over other Resnet sizes is motivated through testing the performance of different sizes of Resnets on the different concentrations of Nocadozole/untreated cells, on which Resnet101 consistently shows the highest performance on the 4 highest concentrations, as shown in Figure 1.

Table 2. *Mean and standard deviation of top1 accuracy from Resnet18 using 12 self-supervised approaches on Cifar10, including VicReg, DeepCluster v2, SWAV, and others, are shown. The experiment uniformly samples 20 hue transformation probabilities with fixed intensity (0.5), keeping other parameters constant. Results show consistent accuracy across methods with minimal standard deviation.*

	Barlow Twins	Byol	Deep Cluster v2	MoCo V2+	nnByol	nnclr	Ressl	SimCLR	SimSiam	SwaV	Vibcreg	Vicreg
Mean	89.59	92.09	86.9	**92.37**	91.3	89.76	90.21	90.16	89.6	86.96	82.47	89.82
Std	0.73	**0.37**	1.9	0.44	0.57	0.74	0.85	0.87	1.01	1.2	0.89	0.94

For the clusterings in Figure 6, we train the same architecture with the same hyperparameters on different compositions of transformations, and perform a Kmeans (K=4) on the resulting representations of the test set. For all the clusters, the images displayed are the images closest to the centroïd of each cluster using an euclidean distance. The clustering in Figure 6 *left* is achieved by using a composition of color jitter with a brightness, contrast and saturation intensity of 0.4, and a hue intensity of 0.125, with a 100% probability, and horizontal and vertical flips, each with 50% probability of application, as

well as random rotations with a maximal angle of 360°, an affine transformation composed of a rotation with an angle of 20°, a translation of 0.1 and a shear with a 10° angle, and a random crop with a scale sampled in the range [0.9;1.1], followed by a resizing of the image to the original size. The clustering in Figure 6 *right* is achieved by color jitter with a brightness, contrast and saturation intensity of 0.4, and a hue intensity of 0.125, with a 100% probability, horizontal and vertical flips, each with 50% probability of application, random rotations with a maximal angle of 360°, and a center crop with a scale of 0.5, followed by a resizing of the image to the original size. For the clustering in Figure **??**, we trained the model with a sum of the losses (and corresponding transformations) of the models used in Figure 6. Through a gridsearch hyperparameter optimization, with the goal of optimizing the AMI score of a Kmeans clustering (K=2), we attributed a coefficient of 0.4 to the loss of the model used in Figure 6 *left*, and a coefficient of 1.0 to the loss of the model used in Figure 6 *right*.

C. Additional results

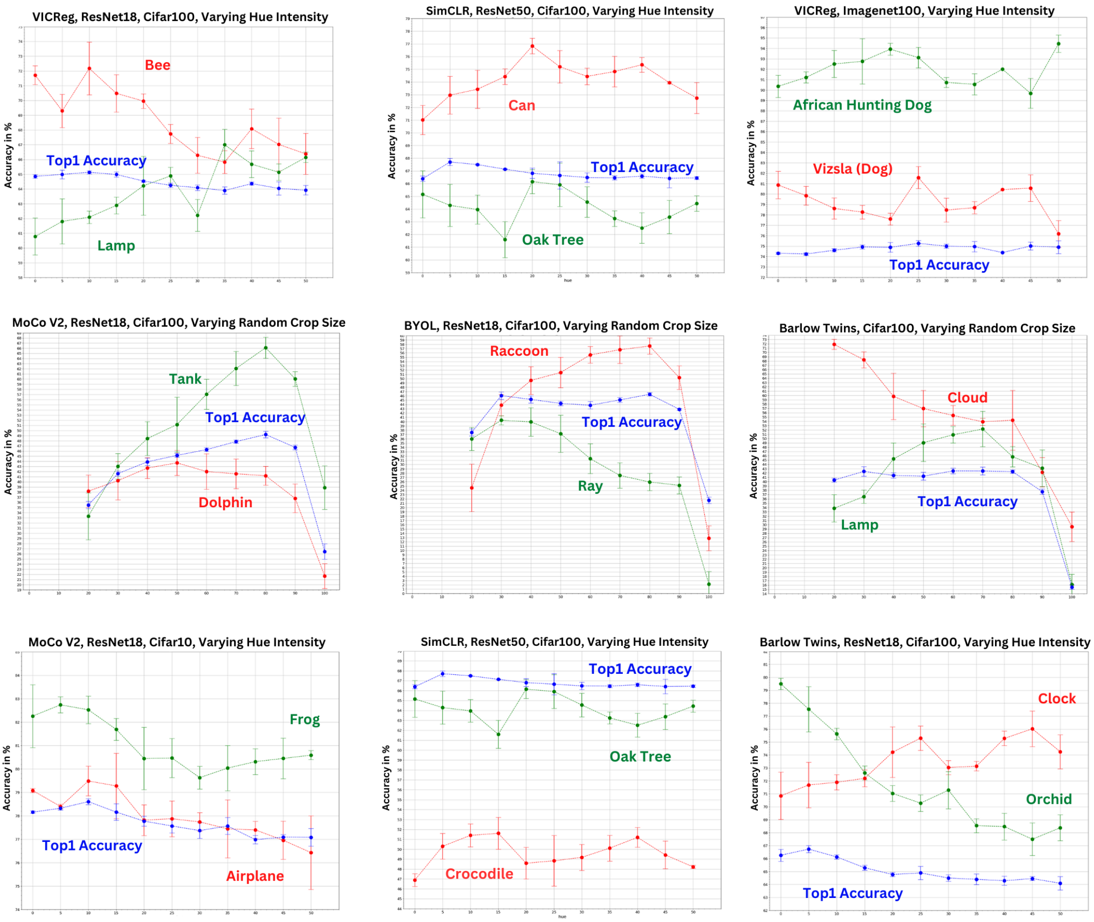

Figure 3. *Resnet architectures' inter-class accuracy on Cifar10, Cifar100, and Imagenet100, trained with various SSRL methods, varying image transformation parameters. Each dot represents the mean and error bar the standard deviation from five runs with different seeds. Results show consistent overall accuracy but reveal transformations' subtle, significant effects on specific class performance.*

Table 3. *Inter-class Bias in Shared Classes Across SSL Methods. The table displays shared class biases in Barlow Twins, BYOL, MoCo v2, SimCLR, and VICReg, trained with a ResNet18 on Cifar100, focusing on Hue Intensity, Color Jitter Probability, and Crop Size. Results underline the impact of transformations on shared biases, confirming class-level biases vary with transformations and SSL methods.*

**Number of shared classes with inter-class bias**	**Hue Intensity**	**Color Jitter Probability**	**Crop size**
In all 5 SSL approaches	51	0	0
In a minimum of 3 SSL approaches	97	3	4
In a minimum of 2 SSL approaches	99	27	8

Table 4. *Linear evaluation results for VGG11, ResNet18, ConvNeXt-Tiny trained with MoCov2 and BYOL SSRL methods on MNIST, using rotations, crops, flips, and random erasing. The outcomes show consistent patterns across SSRL approaches and architectures, indicating robustness.*

Method	VGG11	ResNet18	ConvNeXt-Tiny
BYOL	61.3	62.5	51.6
MoCov2	62.1	63.8	58.7

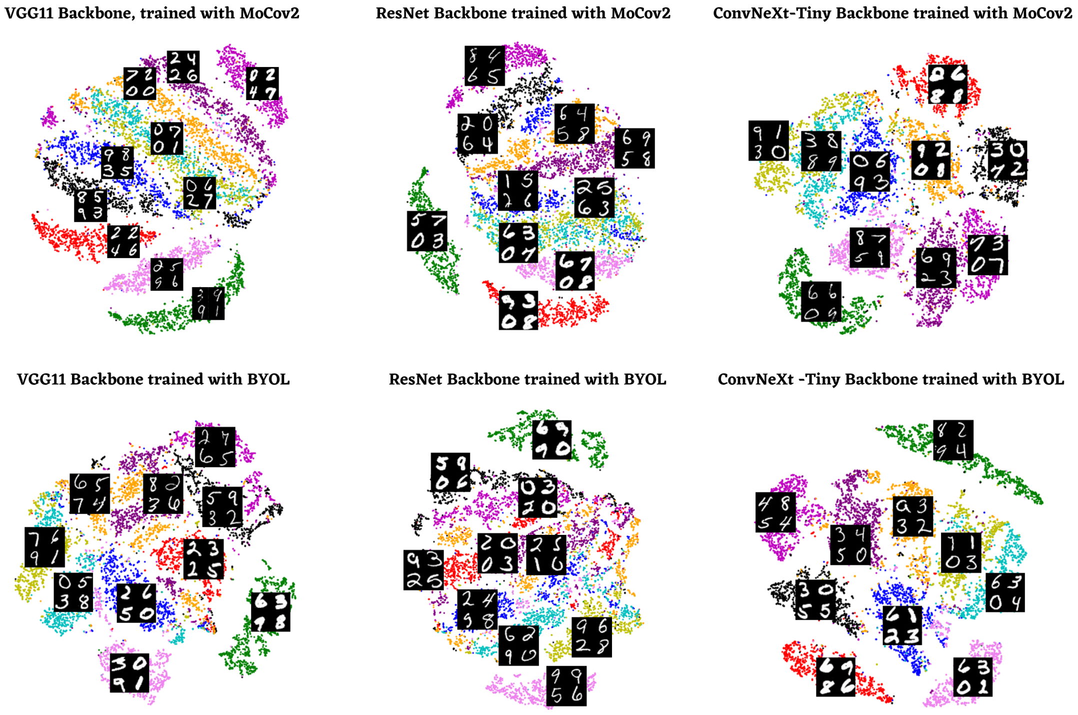

Figure 4. *Clustering results of MNIST dataset using various backbones trained with BYOL and MoCov2 as SSRL approaches, using specific image transformations that preserve the handwriting style and line thickness.*

References

1. Bardes, A., Ponce, J., and LeCun, Y. (2022). Vicreg: Variance-invariance-covariance regularization for self-supervised learning. In *ICLR*.
2. Caie, P. D., Walls, R. E., Ingleston-Orme, A., Daya, S., Houslay, T., Eagle, R., Roberts, M. E., and Carragher, N. O. (2010). High-Content Phenotypic Profiling of Drug Response Signatures across Distinct Cancer Cells. *Molecular Cancer Therapeutics*, 9:1913–1926.
3. Caron, M., Bojanowski, P., Joulin, A., and Douze, M. (2018). Deep clustering for unsupervised learning of visual features. In *ECCV*.
4. Caron, M., Misra, I., Mairal, J., Goyal, P., Bojanowski, P., and Joulin, A. (2020). Unsupervised learning of visual features by contrasting cluster assignments. In *NeurIPS*.
5. Caron, M., Touvron, H., Misra, I., Jégou, H., Mairal, J., Bojanowski, P., and Joulin, A. (2021). Emerging properties in self-supervised vision transformers. In *ICCV*.
6. Chen, T., Kornblith, S., Norouzi, M., and Hinton, G. (2020a). A simple framework for contrastive learning of visual representations. In *ICML*.
7. Chen, X., Fan, H., Girshick, R., and He, K. (2020b). Improved baselines with momentum contrastive learning.
8. Chen, X. and He, K. (2021). Exploring simple siamese representation learning. In *CVPR*.
9. Deng, J., Dong, W., Socher, R., Li, L.-J., Li, K., and Fei-Fei, L. (2009). Imagenet: A large-scale hierarchical image database. In *2009 IEEE conference on computer vision and pattern recognition*, pages 248–255. Ieee.
10. Dwibedi, D., Aytar, Y., Tompson, J., Sermanet, P., and Zisserman, A. (2021). With a little help from my friends: Nearest-neighbor contrastive learning of visual representations. ICCV.
11. Grill, J.-B., Strub, F., Altché, F., Tallec, C., Richemond, P., Buchatskaya, E., Doersch, C., Avila Pires, B., Guo, Z., Gheshlaghi Azar, M., Piot, B., kavukcuoglu, k., Munos, R., and Valko, M. (2020). Bootstrap your own latent - a new approach to self-supervised learning. In *NeurIPS*.
12. Krizhevsky, A. (2009). Learning multiple layers of features from tiny images.
13. Kuhn, H. W. (1955). The hungarian method for the assignment problem. *Naval Research Logistics Quarterly*, 2(1-2):83–97.
14. LeCun, Y., Cortes, C., and Burges, C. J. (1998). The mnist database of handwritten digits.
15. Lee, D. and Aune, E. (2021). Computer vision self-supervised learning methods on time series.
16. Ljosa, V., Sokolnicki, K. L., and Carpenter, A. E. (2012). Annotated high-throughput microscopy image sets for validation. *Nature Methods*, 9:637–637.
17. Simonyan, K. and Zisserman, A. (2015). Very deep convolutional networks for large-scale image recognition. In *ICLR*.
18. Vinh, N. X., Epps, J., and Bailey, J. (2010). Information theoretic measures for clusterings comparison: Variants, properties, normalization and correction for chance. *Journal of Machine Learning Research*, 11:2837–2854.
19. Zbontar, J., Jing, L., Misra, I., LeCun, Y., and Deny, S. (2021). Barlow twins: Self-supervised learning via redundancy reduction. In *ICML*.
20. Zheng, M., You, S., Wang, F., Qian, C., Zhang, C., Wang, X., and Xu, C. (2021). Ressl: Relational self-supervised learning with weak augmentation. NeurIPS.
